# Preliminary evaluation of exome sequencing to identify genetic markers of susceptibility to tuberculosis disease

**DOI:** 10.1186/s13104-015-1740-5

**Published:** 2015-12-08

**Authors:** Carla Duncan, Frances Jamieson, Carolina Mehaffy

**Affiliations:** Public Health Ontario, Toronto, ON Canada; Department of Laboratory Medicine and Pathobiology, University of Toronto, Toronto, ON Canada; Department of Microbiology, Immunology and Pathology, Colorado State University, 1619 Campus Delivery, Fort Collins, CO 80523-1601 USA

**Keywords:** Exome sequencing, Single nucleotide polymorphisms, Tuberculosis

## Abstract

**Background:**

Recent studies have shown that certain human genetic polymorphisms could be associated with susceptibility to tuberculosis (TB) infection and disease. Advances in next generation sequencing include the ability to rapidly sequence the entire human exome. These new technologies can be exploited to identify new associations of human genetic polymorphisms and TB infection and disease. In this preliminary study we compared two different strategies for sequencing of the human exome in a small sample set consisting of three individuals with a history of TB disease and two individuals with latent TB infection.

**Findings:**

Sequencing of the entire exome of the five participants using Agilent SureSelect kit resulted in the identification of 1611 single nucleotide polymorphisms (SNPs) that were only present in the individuals with a history of active TB but not in the latent TB cases. Alternatively, sequencing of 4000 target genes available in the TruSight kit resulted in identification of 182 SNPs only present in the active TB cases and not in the latent TB participants. The overlap of the two kits was 112 SNPs.

**Conclusions:**

Even though this pilot study was restricted to a small number of participants, we demonstrated the feasibility of using exome sequencing technologies to mine potential genetic associations of susceptibility to TB disease and presented a number of potential targets that can be further explore in larger research trials.

**Electronic supplementary material:**

The online version of this article (doi:10.1186/s13104-015-1740-5) contains supplementary material, which is available to authorized users.

## Findings

### Rationale

Recent studies have shown that certain human genetic polymorphisms could be associated with susceptibility to TB [1–10]. Many of the tested genes identified to-date are involved directly or indirectly with immune and inflammation mechanisms [4–10]. However, it is possible that other polymorphisms outside of these target genes may be involved in susceptibility to TB. Recent advances in sequencing technologies and tools have made it possible to rapidly sequence the entire coding region of the human genome (exome) and allowing the identification of novel polymorphisms associated with a particular disease, disorder or condition [11–14]. Exome sequencing has been exploited to evaluate gene variants associated with certain cancers, however, its use in the study of susceptibility to infectious diseases is still limited [15, 16]. Tuberculosis infection and disease has a long history of association with the human race [17, 18] and given its intricate relationship with the host, it is not surprising that certain genes and gene variants or mutations have been thought to be involved in the ability of the host to fight the infection and/or halt its progression to disease.

### Goal

The main focus of this pilot study was to evaluate the utility of two different kits for exome sequencing as a means to identify novel polymorphisms associated with susceptibility to TB. In Toronto, Canada, the largest clusters of active TB transmission are associated with the under housed or homeless population [19–21] and therefore another goal of the study was to evaluate the feasibility to recruit participants in this group, including individuals with past history of TB disease as well as individuals with a record of TB infection.

### Ethics, consent and permission

Ethics approval for this study was granted by the institutional Ethics Research Board at Public Health Ontario, file number 2013-038. All participants received and signed an informed consent to voluntarily participate in this study.

### Methods

After ethics approval was obtained, we conducted 1 month of recruitment at one of Toronto’s inner city shelters. In total we recruited nine individuals. Three of these had a positive history of active TB disease for which the genotyping data indicated their infection was due to either one of the four *M. tuberculosis* strains previously associated with this population [19, 22]. The remaining six individuals were recruited as potential candidates for the latent TB control group, however, only two of them had a record of a positive TB skin test indicating TB latent infection. The four individuals with no proven record of TB infection were not enrolled in the study. Blood samples were obtained from all participants and used to extract gDNA using the Qiagen QIAamp DNA blood mini kit as per the manufacture’s protocol. Two different sequencing kits were evaluated, Agilent SureSelect^XT^ Target Enrichment System—Human All Exon V5 + UTRs and Illumina TruSight™ One. Illumina TruSight™ One targets 4813 genes, including several immune related genes previously shown to be involved in TB infection and or host–pathogen interactions. gDNA from all participants was used to prepare the libraries for each kit as recommended by the manufacturer. SureSelect libraries were pooled and sequenced in two lanes of an Illumina Hiseq 2500 instrument at the Clinical Genomics Centre in Mount Sinai Hospital and TruSight libraries were pooled in groups of three and sequenced in an Illumina MiSeq at our institutional genomics core. Data analysis was performed using the CLC Genomics Workbench v7.0 software by mapping the reads to the reference Human genome hg19 (GRCh37 Ensemble 74). Single nucleotide variants were identified using a quality based variant detection with a minimum coverage cutoff of 10× and a minimum frequency of 35 % for heterozygous alleles. Variants were annotated using the dbSNP database (http://www.ncbi.nlm.nih.gov/snp/). Only SNPs identified in the target regions and in all three active TB cases, and absent in the two latent TB cases, were kept for analysis.

### Outcomes

The total number of targets covered with at least a 10× coverage ranged from 94.6 to 96 % for the SureSelect and 93.8–94.9 % for the TruSight kit. The two enrichment kits performed relatively similar in terms of time and cost of library preparation and sequencing costs. Promoter regions located outside of the untranslated regions (UTRs) are not covered in any of the two kits and therefore potential polymorphisms in these regions are not identified. However, given the large number of exon regions covered by the SureSelect, this kit is ideal to use in a discovery assay for the identification of novel polymorphisms associated to a given condition.

Using the SureSelect kit we identified 1611 SNPs that were only present in the Active TB cases. Using the TruSight kit we identified 182 SNPs only present in the active TB cases and not in the latent TB participants. The overlap of the two kits was 112 SNPs (Additional file 1: Table S1). The 70 SNPs found in the TruSight kit data that did not overlap with the SureSelect kit were examined in more detail. Of these, 21 were in target regions not covered by the SureSelect kit, 15 were not called in the SureSelect data because the ratio of alleles was <35 %, 26 were in found in the latent cases but not called in the TruSight data due to insufficient coverage, six were not called in the SureSelect data due to insufficient coverage and two were incorrect calling due to stretches of homopolymers.

Since TB is an infectious disease with a complex interaction between host, pathogen and environmental factors [23], it is possible that multiple genetic variants in the host may be involved in susceptibility to disease as opposed to rare genetic disorders that are often explained by single variants [1]. A gene ontology (GO) enrichment analysis was performed in order to identify possible pathways, as opposed to single variants, that may be involved in susceptibility to TB. When the 1611 SNPs identified in active TB cases only using the SureSelect kit were analyzed, several molecular pathways were identified as being significantly enriched (*p* value <0.01), including some with a known link to TB infection. These included (1) cellular response to ATP and purinergic nucleotide receptor signaling pathway; (2) apoptotic processes; and (3) vitamin metabolic and biosynthetic processes (Table 1). These pathways were also identified when only the 112 SNPs that overlap with the two kits were analyzed (data not shown).

**Table 1:** Gene ontology (GO) enrichment analysis of 1611 SNPs identified at 10× coverage using SureSelect kit with P < 0.01

GO description	Occurrences		P values	Link to tuberculosis
	All genes	Sample		
Cellular response to ATP	7	4	3.23E−4	[41, 42]
Riboflavin metabolic process	5	3	1.71E−3	
Regulation of centriole replication	5	3	1.71E−3	
Axon guidance	281	28	2.98E−3	
Vitamin E metabolic process	2	2	3.27E−3	[43]
Epithelial cell proliferation	2	2	3.27E−3	
Response to cortisol stimulus	2	2	3.27E−3	
Negative regulation of retinal ganglion cell axon guidance	2	2	3.27E−3	
Cellular response to hormone stimulus	12	4	3.63E−3	
O-glycan processing	55	9	3.71E−3	
Regulation of small GTPase mediated signal transduction	121	15	3.75E−3	
Integrin-mediated signaling pathway	37	7	4.45E−3	
Purinergic nucleotide receptor signaling pathway	7	3	5.49E−3	[44]
Synaptic vesicle endocytosis	7	3	5.49E−3	
Negative regulation of phosphatase activity	49	8	6.18E−3	
Response to unfolded protein	30	6	6.26E−3	[45]
Autophagic vacuole fusion	8	3	8.41E−3	[46]
Nucleocytoplasmic transport	15	4	8.73E−3	
Small GTPase mediated signal transduction	158	17	8.93E−3	
Pre-B cell allelic exclusion	3	2	9.43E−3	
Pressure natriuresis	3	2	9.43E−3	
Chemorepulsion involved in embryonic olfactory bulb interneuron precursor migration	3	2	9.43E−3	
Positive regulation of telomere maintenance via telomerase	3	2	9.43E−3	
Lacrimal gland development	3	2	9.43E−3	
Roundabout signaling pathway	3	2	9.43E−3	
Leukotriene B4 catabolic process	3	2	9.43E−3	
Vitamin K biosynthetic process	3	2	9.43E−3	
Metaphase plate congression	3	2	9.43E−3	
Regulation of endopeptidase activity	3	2	9.43E−3	
Apoptotic process involved in luteolysis	3	2	9.43E−3	

We also interrogated our data against a previously reported list of variants in 26 innate immune genes that have been shown to be associated with susceptibility to TB [4]. For this analysis we used the entire SSTE SNP data set that the met the quality standards outlined above. We included variants identified in intron or intergenic regions only if sufficient coverage (10×) was present in all five samples. We identified SNPs in several innate immune genes previously shown to be potentially associated with active TB [4] (Table 2). Based on the SNPs identified in active TB subjects, three of these genes appear to be related to active TB in our study (i.e. TLR1, TNF and VDR). Although we identified SNPs in the remaining genes listed in Table 2, these were found in both latent and active TB cases or only in individuals with latent TB. Of the three genes with SNPs present in active TB cases but not in latent cases: toll-like receptor-1 (TLR1), vitamin D receptor (VDR) and tumor necrosis factor (TNF), only SNPs in TLR1 were found in all three active cases. SNPs in these three genes were also confirmed by Sanger sequencing (Additional file 2). Both the A743G (Asn148Ser) and G1805T (Ser602Ile) variants were identified in TLR1. Although data regarding a link between TLR1 variants and susceptibility to TB is ambiguous [24–27], it has been shown that the 602Ser allele inhibits cell surface trafficking leading to a lack of TLR1 on the plasma membrane and a hypo-responsiveness to TLR1 agonists including mycobacterial membrane preparations [24, 25, 28]. The role of VDR and susceptibility to TB is also under debate [29, 30] but the active metabolite of vitamin D (1,25-dihydroxyvitamin D3) suppresses the growth of *M. tuberculosis* in vitro [31, 32] and vitamin D deficiency has been associated with susceptibility to active TB [33]. TNF is a cytokine that plays a key role in granuloma formation [34–36]. Neutralization of TNF leads to a lack of control of initial or chronic infection and loss of granuloma structure. There have been multiple conflicting studies examining the role of SNPs in the TNF promoter at the −308 position and TB susceptibility [37–40] however there is no evidence that SNPs in this region effect the transcription of TNF [36]. Conflicting results is not surprising in a complex diseases like TB, where many gene variants could be interacting to result in disease, and the combination of different gene variants could lead to the same outcome.

**Table 2:** Details of SNPs found in innate immune genes at 10× coverage using SSTE kit which have been associated with susceptibility to TB [4]

Gene	Polymorphism SNP	Coding change	Amino acid change	Genetic location	Active cases	Latent cases
TLR1	rs4833095	743A > G	Asn248Ser	Exon	3	0
	rs5743618	1805G > T	Ser602Ile	Exon	3	0
TLR8	rs3764880	1A > G	Met1Val		2	1
TLR9	rs352143	169A > G	Asn57Asp		1	1
TIRAP	rs8177374	539C > T	Ser180Leu		0	1
NOD2	rs2066842	802C > T	Pro268Ser	Exon	0	1
	rs2066844	2104C > T	Arg702Trp	Exon	0	1
VDR	rs2228570	2T > C	Met1		3	2
	rs731236 (*TaqI*)	1056T > C		Coding-synon	1	0
ILIB	rs1143634	315C > T		Coding-synon	0	1
TNF	rs1800629	−308G/A		Promoter	1	0

Multiple SNPs that have not been previously reported to be associated with susceptibility to TB were found in all of these genes

### Impacts

The work outlined in this study was completed in 16 months. This included review by the Ethics Board, training of the research nurse to recruit potential participants in one of Toronto’s under-housed shelters, recruitment, sample collection, sequencing and data analysis. Active recruitment of participants at the shelter site was completed in only 1 month. However, even in that short period of time we were able to recruit nine potential participants and we anticipate that larger trials with an expansion in duration of recruitment as well as number of targeted shelters will result in a much larger participation by individuals that meet the recruitment specifications. Even though our pilot study only included three individuals with a history of active TB and two individuals with confirmed TB infection but not disease, exome sequencing proved to be a powerful technique to identify potential host genetic variants associated with susceptibility to TB disease. Future studies expanding the number of individuals in each cohort will help narrow down the number of potential targets for validation.

To our knowledge, this is the first study that has used exome sequencing to potentially identify genomic markers of susceptibility to TB resulting in a large set of data including potential single nucleotide variants as well as molecular and cellular pathways that may be associated with TB infection and disease.

## Additional files

10.1186/s13104-015-1740-5 Materials and methods for PCR and Sanger sequencing of selected SNPs.
10.1186/s13104-015-1740-5 List of SNPs identified using both sequencing kits (overlapping SNPs).
