# Supplementary material for: Preliminary evaluation of exome sequencing to identify genetic markers of susceptibility to tuberculosis disease
Source: BMC Res Notes. 2015 Dec 8;8:750. doi: 10.1186/s13104-015-1740-5 (PMC4672511; doi:10.1186/s13104-015-1740-5)
Supplement: Supplementary file 2 — 10.1186/s13104-015-1740-5 List of SNPs identified using both sequencing kits (overlapping SNPs). [file 13104_2015_1740_MOESM2_ESM.docx]

Supplementary Methods

PCR and Sequencing:

*Taq* DNA polymerase, deoxynucleoside triphosphates, and buffer were used according to the recommendations of the manufacturer (Invitrogen, Burlington, ON, Canada). A list of primers used can be found in Table S1. Sequencing reactions were performed using a BigDye terminator cycle sequencing kit, version 3.1, purified using a BigDye X terminator purification kit, and run on a 3130xl genetic analyzer (Applied Biosystems, Streetsville, ON, Canada)

Table S1: primers for verifying SNPs

| gene | SNP | Forward Primer | Reverse Primer | Amplicon  Size (bp) |
| --- | --- | --- | --- | --- |
| TLR1 | rs4833095 | TGTCAGTCAAGACTGTAGCAAA | TCCAGCTGACCCTGTAGCTT | 241 |
| TLR1 | rs5743618 | AGAGGAACCCTACTAAAGGACT | TTCACCCAGAAAGAATCGTGC | 269 |
| VDR | rs731236 | TGAGAGCTCCTGTGCCTTCTT | ACTGCTTGGAGTGCTCCTCATTG | 218 |
| TNF | rs1800629 | TTCTGAAGCCCCTCCCAGTT | TACCCCTCACACTCCCCAT | 224 |
